# Loss of Pseudouridine Synthases in the RluA Family Causes Hypersensitive Nociception in *Drosophila*

**DOI:** 10.1534/g3.120.401767

**Published:** 2020-12-01

**Authors:** Wan Song, Susanne Ressl, W. Daniel Tracey

**Affiliations:** * Gill Center for Biomolecular Research, Indiana University, Bloomington, IN 47405; † Department of Neuroscience, University of Austin Texas, Austin, TX 78712; ‡ Department of Biology, Indiana University, Bloomington, IN 47405

**Keywords:** *Drosophila melanogaster*, pseudouridine, pseudouridine synthase, nociception, behavior, hyperalgesia, pain, dendrite, sensory neuron

## Abstract

Nociceptive neurons of *Drosophila melanogaster* larvae are characterized by highly branched dendritic processes whose proper morphogenesis relies on a large number of RNA-binding proteins. Post-transcriptional regulation of RNA in these dendrites has been found to play an important role in their function. Here, we investigate the neuronal functions of two putative RNA modification genes, *RluA-1* and *RluA-2*, which are predicted to encode pseudouridine synthases. *RluA-1* is specifically expressed in larval sensory neurons while *RluA-2* expression is ubiquitous. Nociceptor-specific RNAi knockdown of *RluA-1* caused hypersensitive nociception phenotypes, which were recapitulated with genetic null alleles. These were rescued with genomic duplication and nociceptor-specific expression of *UAS-RluA-1-cDNA*. As with *RluA-1*, *RluA-2* loss of function mutants also displayed hyperalgesia. Interestingly, nociceptor neuron dendrites showed a hyperbranched morphology in the *RluA-1* mutants. The latter may be a cause or a consequence of heightened sensitivity in mutant nociception behaviors.

Pain serves an indispensable, protective role but when pain becomes pathological it can have a debilitating impact on human life. The total annual cost of pain to society in the United States was estimated by the Institute of Medicine to be up to $635 billion, which is greater than that of heart disease, cancer, and diabetes combined (Gaskin and Richard 2012). It is therefore of urgent importance to uncover the basic molecular and cellular mechanisms involved in pain in order to better treat it. Laboratory animal models of pain and nociception have played an essential role in identifying such mechanisms. *Drosophila* larvae respond to noxious thermal and mechanical stimuli through stereotyped rolling escape locomotion (in which the larva rotates around its long body axis) which is easily distinguishable from other forms of locomotion (Tracey *et al.* 2003). Combined with the unparalleled genetic tools available for *Drosophila melanogaster*, this behavioral readout provides an excellent system to study the genetics of nociception and pain (Tracey *et al.* 2003; Caldwell and Tracey 2010; Milinkeviciute *et al.* 2012; Im and Galko 2012; Tracey 2017; Khuong *et al.* 2019). Previous studies have demonstrated a specific subset of dendritic arborization (da) sensory neurons in the peripheral nervous system, Class IV multidendritic da (cIVda) neurons, are of critical importance for thermal, mechanical, and high intensity light nociception (Grueber *et al.* 2002; Hwang *et al.* 2007; Xiang *et al.* 2010). Further evidence suggests a lesser but significant contribution of Class II (cIIda) and Class III da (cIIIda) neurons in mechanical nociception (Hwang *et al.* 2007; Kim *et al.* 2012; Hu *et al.* 2017). In addition, great progress has been made in identifying the circuits in the larval abdominal ganglion that are involved in rolling escape locomotion (Ohyama *et al.* 2013; Ohyama *et al.* 2015; Chin and Tracey 2017; Burgos *et al.* 2018).

Genes that are specifically expressed in multidendritic (md) neurons have been found to play a role in nociception. For instance, *painless* (Tracey *et al.* 2003) is required for mechanical and thermal nociception and it is expressed in all four classes of md neurons. Similarly, *straightjacket* (Neely *et al.* 2010) is expressed in md neurons and required for avoidance of noxious heat. Mechanical nociception genes such as *pickpocket* (Zhong *et al.* 2010), *ppk26*/*balboa* (Mauthner *et al.* 2014; Gorczyca *et al.* 2014; Guo *et al.* 2014) and the polymodal nociception gene *dTRPA1-C/D* (Zhong *et al.* 2012) each show very specific expression in the cIVda neurons. Forward genetic screens have also identified a set of genes with enriched expression in the cIVda neurons which either inhibit or activate nociceptive pathways (Honjo *et al.* 2016).

The historically first known genetic marker with specific expression pattern in the da neurons was the *lacZ* enhancer trap *E7-2-36* (Brewster and Bodmer 1995). Later studies reported that this enhancer trap gene was inserted upstream of the *RluA-1* gene and DNA sequences from upstream of *RluA-1* caused expression of GAL4 in multidendritic neurons (Wang *et al.* 2011). The *RluA-1* gene encodes an enzyme that is predicted to have the conserved pseudouridine synthase domain required to catalyze the isomerization of uridines to pseudouridines on RNA (Sivaraman *et al.* 2004; Wang *et al.* 2011; Waterhouse *et al.* 2018), but how this RNA modifying protein is involved in the function of nociceptive multidendritic neurons remains unknown.

A widespread importance of RNA-binding proteins in cIVda neuron dendrite morphogenesis and function was found in a recent large-scale RNAi screen that identified 88 genes encoding RNA-binding proteins whose knockdown caused aberrant dendrite morphogenesis (Olesnicky *et al.* 2014). The elaborate dendrite arbors of cIVda neurons project long distances from the neuronal cell body and mRNA granules are trafficked to these distant sites where they may undergo local translation. Indeed, RNA granules containing Nanos (Nos), Pumilio (Pum), Oskar (Osk), Fragile X Mental Retardation (FMRP) and other proteins have been shown to regulate the formation of higher order dendrites in these cells (Ye *et al.* 2004; Pan *et al.* 2004; Brechbiel and Gavis 2008; Bianco *et al.* 2010; Xu *et al.* 2013). Mechanical nociception defects are also observed in animals with disruption in these pathways (Xu *et al.* 2013).

Pseudouridylation is the most common post-transcriptional RNA modification. Pseudouridine (Psi, Ψ), the C5-glycoside isomer of uridine, was initially found in many positions in rRNA, tRNA and snRNA in all organisms that have been investigated (Ge and Yu 2013). RNA-seq based global pseudouridine profiling has shown the presence of Ψ in many mRNAs and a large number of those sites were found to be dynamically regulated in yeast and human cells (Carlile *et al.* 2014; Lovejoy *et al.* 2014; Schwartz *et al.* 2014; Khoddami *et al.* 2019). Dysfunctional pseudouridylation has been linked to several human diseases (Knight *et al.* 1999; Fujiwara and Harigae 2013; de Brouwer *et al.* 2018). Since many sites of pseudouridylation in different organisms are evolutionarily conserved, *Drosophila melanogaster* provides an excellent and genetically tractable metazoan system to elucidate some of these functions (Giordano *et al.* 1999; Deryusheva and Gall 2013; de Brouwer *et al.* 2018).

The isomerization of uridine to pseudouridine is catalyzed by six families of pseudouridine synthases. They function either as guide RNA directed ribonucleoprotein complexes or as stand-alone proteins (Koonin 1996; Hamma and Ferré-D’Amaré 2006). In the *Drosophila* genome, 9 proteins have been identified with annotated pseudouridine synthase domains. Minifly (mfl), the RNA-dependent pseudouridine synthase homolog of human dyskerin (mouse NAP57 and yeast Cbf5), is required for somatic stem cell homeostasis and is essential for *Drosophila* viability and fertility (Phillips *et al.* 1998; Giordano *et al.* 1999; Vicidomini *et al.* 2017). Knockout of *Drosophila* Pus7, the human and yeast Pus7 homolog, results in increased aggressiveness in adult flies (de Brouwer *et al.* 2018). The function and specificity of other predicted pseudouridine synthases are largely unknown. Among the six families, the RluA family, which does not rely on guide RNAs, appears to be the most complex based on divergent substrate specificities in bacteria and yeast (Hoang *et al.* 2006). RluA family members in bacteria are involved in ribosomal assembly and growth (Raychaudhuri *et al.* 1999; Gutgsell *et al.* 2005) but their function in multicellular organisms has not been studied. Although pseudouridine synthases appear to function ubiquitously, as noted above, *Drosophila **RluA-1*, a member in RluA family, has been reported to be specifically expressed in md neurons (Wang *et al.* 2011). Thus, we have investigated the role for *RluA-1* and its paralog *RluA-2*, in nociception pathways that are known to depend on md neurons. Our results indicate an important function for *RluA-1* and *RluA-2* in the regulation of nociception.

## Materials and Methods

### Fly strains and husbandry

The following fly strains were obtained from Bloomington Stock Center: (*w^1118^*; *PBac{vas-Cas9}VK00027*), *Mi{MIC}RluA-1^[MI06897]^*, *Mi{MIC}RluA-2^[MI12981]^*, *Df(2L)Exel7048/CyO*, (*yw; Sp/CyO;* *pC-(lox-attB2-SA-T2A-Gal4-Hsp70)3*), (*yw hs-cre, vasΦC31; Sp/CyO;* Sb/TM3Ser), (*P{ry=hsFLP}, yw M{vas-int.B}ZH-2A;* *Sp/CyO;* *P{FRT-attB-{GFSTF}-attB(w+)-FRT}*), *ppk-CD4-tdTom*, *md-Gal4*, *UAS-mCD8::RFP,* *40XUAS-mCD8::GFP*. The following fly strains were obtained from Exelixis collection at Harvard Medical School: *PBac{WH}^f02750^*,* P{XP}d2586, PBac{WH}Grip75^[f05483]^*, *PBac{WH}RluA-2^[f07702]^*. The RNAi lines targeting *RluA-1* (31719-R1) were obtained from Kyoto Stock Center. The genetic duplication line (BAC ID: CH321-49P21) covering *RluA-1* (starting at 24,819,420 and ending at 24,910,132 on 2R) was obtained from Genetivision. The *nos-Cas9* line used for generating *RluA-2^del-HDR^* and double mutant *RluA-1^del-HDR^RluA-2^del-HDR^* [*y sc v; {nos-Cas9} attP2 (TH00787.N*)] was kindly provided by the Norbert Perrimon lab. All larvae used in experiments were reared on the Bloomington Drosophila medium in an incubator with controlled temperature (25°) and humidity (70%) on a 12h light/12h dark cycle. Strains were otherwise maintained at room temperature.

### Multiple sequence alignment and homology model

The predicted protein sequences of RluA-1 and RluA-2 from *Drosophila melanogaster* (Dm) were aligned with their closest homologs from other model organisms, including RUSD2 (*Homo sapiens*, Hs), RUSD2 (*Mus musculus*, Mm), RIB2 and its paralog PUS9 (*Saccharomyces cerevisiae*, Sc), PUS7 (*Arabidopsis thaliana*, At), RluA, RluC, RluD and RsuA (*Escherichia coli*, Ec). Entrez database accession numbers are as follows: RluA-1-PABC_Dm: Q9VKV0, RluA-2-PC_Dm: Q9VKU8, RUSD2_Hs: Q8IZ73, RUSD2_Mm: Q149F1, RIB2_Sc: Q12362, PUS9_Sc: Q12069, PUS7_At: F4KBV6, RluA_Ec: P0AA37, RluC_Ec: P0AA39, RluD_Ec: P33643, RsuA_Ec: P0AA43. The sequences were aligned using the Tcoffee multiple sequence alignment tool M-coffee (http://tcoffee.crg.cat/apps/tcoffee/do:mcoffee) with default parameters. Resulting alignment was rendered in ESPript (Robert and Gouet 2014) using secondary structure information of *E. coli* RluA to produce visualization. The structural model of the pseudouridine synthase domain of RluA-1 (isoform A, B and C) and RluA-2 (isoform C) in *D. melanogaster* were generated using ModBase (https://modbase.compbio.ucsf.edu) (Pieper *et al.* 2014). The model for RluA-1 was based on the RluA *E. coli* structure PDB ID 2i82 chain A, covering amino acid sequence 365-554 with a GA341score of 1, a moderate MPQS score of 0.62 due to moderate sequence identity E-Value of 28%, a TSVMod NO35 of 75.6% indicating a high native overlap at 3.5 Å. More model statistics can be found in the provided RluA-1 model pdb file (RluA1_modbase-model_a0f26b38405803a459cd5f2a1b884076.pdb, File S1). The model for RluA-2 was based on the RluC *E. coli* structure PDB ID 1vk9 chain A, covering amino acid sequence 218-439, with a GA341score of 1, a moderate MPQS score of 0.74 despite higher sequence identity E-Value of 32% (compared to above), a TSVMod NO35 of 73.9% indicating a high native overlap at 3.5 Å. More model statistics can be found in the provided RluA-2 model pdb file (RluA2_modbase-model_f4f7b951d5f80383ba7f298dcc585bb0.pdb, File S2). Structures were analyzed and visualized using PyMol (The PyMOL Molecular Graphics System, Version 2.0, Schrödinger, LLC).

### RNA isolation, RT and Q-PCR analysis

To evaluate the *RluA-1*-RNAi efficiency, a stock of the *md-Gal4* driver built with *UAS-dicer2* (*w*; *md-GAL4*; *UAS-dicer2*) was crossed to *UAS-RluA-1-RNAi* (test) or its genetic background (*w^1118^*, control). A batch of four L3 larvae were rinsed in 1xPBS and quickly frozen in liquid nitrogen for RNA extraction. RNA isolation was performed using TRIzol (Life Technologies) following the manufacturer’s protocol. 1 µg of total RNA samples were subjected to DNase treatment and reverse-transcription using the SuperScript IV reverse transcriptase enzyme and Oligo(dT)_12-18_ primer (Life technologies). cDNA was amplified in real time using the qPCR Master mix plus for power SYBR Green I assay (Invitrogen) and analyzed with the QuantStudio Real-Time PCR System (Applied Biosystems). Intron-spanning primer pairs (RluA-1-cDNA-F: 5′-GAGCAGCAGATTCGCAACAG, RluA-1-cDNA-R: 5′-ACTTCAATGGGCTCCTTGCA) and (Act5C-F: 5′-GGGGCAGAGCAAGCGTGGTA, Act5C-R: 5′-GGGTGCCACACGCAGCTCAT) were used for *RluA-1* and *Actin5C*, respectively. The level of expression for *RluA-1* in RNAi expressing line (*md-Gal4*>*RluA-1-RNAi*) was normalized based on the *Actin5C* and expressed as a percentage of the control.

### CRIPSR targeting of RluA-1 and RluA-2

HDR for mutagenesis used the CRISPR/Cas9 (Gratz *et al.* 2013) to generate *RluA-1*^*del-HDR*^, *RluA-2*^*del-HDR*^ and the double mutant *RluA-1*^*del-HDR*^*RluA-2*^*del-HDR*^. Target sites were selected using the flyCRISPR Optimal Target Finder. For the precise deletion of *RluA-1*, the gRNA target sites are 5′ end 5′-CCACTG∣TGCAGCGGAAAATTCAC-3′ (where “∣” represents the Cas9 cut site, which is 71bp upstream of the *RluA-1* transcriptional start) and 3′ end: 5′-ACATATATTCAAAAGCTCTTTGG-3′ (cut site at 60bp downstream of *RluA-1* 3′UTR). We used the following primer pairs to amplify the 885bp *RluA-1* homology ARM1: *RluA-1*-ARM1F: 5′-AATACACCTGCATTATCGCTGGTCCCTGTGGCTTTGCAC-3′,

*RluA-1*-ARM1R: 5′-AATACACCTGCAATTCTACCAGTGGGGCAAACCGCATTT-3′. We used *RluA-1*-ARM2F: 5′-GACTGCTCTTCGTATCTTTGGATGGTAAGTGCTTAAAC-3′ and *RluA-1*-ARM2R: 5′-ATTAGCTCTTCTGACCTTACAACTCCTTCAAGTCA-3′ to amplify 969 bp of *RluA-1* homology ARM2. For *RluA-2*, we used a target site at the 5′ end: 5′-GCAATCTATAGGTCTGC∣GGAAGC-3′ (cut site at bp 5 of exon 3) and at the 3′ end: 5′AAGATAAACTACAGAGA∣CCCCGG-3′ (cutting site in the middle of exon 8) so that the conserved pseudouridine synthase domain (located in exon 5) would be deleted. Primer pairs *RluA-2*-ARM1F: 5′CTAACACCTGCATATTCGCTCGAAACCCATTGTTAGCTG3′ and *RluA-2* ARM1R: 5′ CATTCACCTGCATTACTACGCAGACCTATAGATTGCAAT3′ were used to amplify the 979bp *RluA-2* homology ARM1. *RluA-2*-ARM2F: 5′CAGTGCTCTTCGTATCCCCGGCACAAAGGATCTCA-3′, and *RluA-2*-ARM2R: 5′ GACTGCTCTTCCGACATTTCAATGCCCTTGGCCAA-3′ were used to amplify the *RluA-2* ARM2 (998bp). Rapid dsDNA donor cloning was carried out with the *pHD-DsRed-attP* vector (Beumer and Carroll 2014) and the guide RNA (gRNAs) were cloned into *pU6-BsbI-chiRNA* vector (Gratz *et al.* 2013). Embryos of *vas-Cas9* on chromosome III (*w^1118^*; *PBac{y[+mDint2]=vas-Cas9}VK00027*, “injection line 1”*)* were injected with *RluA-1* dsDNA donor and gRNAs to generate G_0_ founders for *RluA-1*^*del-HDR*^. Embryos with *nos-Cas9* on chromosome III [*y sc v*; *{nos-Cas9} attP2* (TH00787.N), “injection line 2”] were injected with *RluA-2* dsDNA donor and gRNAs to create the G_0_ founders for *RluA-2*^*del-HDR*^. For generating the double mutant *RluA-1*^*del-HDR*^*RluA-2*^*del-HDR*^, *nos-Cas9* was first introduced to the *RluA-1*^*del-HDR*^ mutant background and the DsRed marker in *RluA-1*^*del-HDR*^ removed with CRE recombinase and the resultant homozygous strain [*yw*; *RluA-1*^*del-HDR*^*ΔDsRed*; *{nos-Cas9}attP2* (TH00787.N), “injection line 3”] was injected with the *RluA-2* dsDNA donor and gRNAs. All embryo injections of the dsDNA donor (at a concentration of 500ng/ul) and gRNAs (100ng/ul for each) were performed by the Model system Injections (modelsysteminjections@flymsi.com).

To identify the desired HDR mutants, G_0_ flies were crossed to *w^1118^* and single F_1_ founders were identified with DsRed fluorescence in the eyes (from *3XP3-DsRed* reporter) and mated with a second chromosome balancer strain to establish independent lines. An initial molecular screening for the desired events was performed by PCR on gDNA extracted from candidates placed over a deficiency (*Df(2L)Exelixis7048*) with genomic primers located outside of the homology arms for *RluA-1* and *RluA-2*, respectively (RluA-1-front-F: 5′-GAGTAATTGTGGGTGTGCCAGAG-3′ and RluA-1-end-R: 5′-CTGGACTTTTGTTACCCCTT-3′ *for **RluA-1*, RluA-2-front-F: 5′- CGGATTGGAAATGTGCCATC -3′ and RluA-2-end-R: 5′- TTCCAGTTGAATATCGCCGTG -3′ for *RluA-2*, data not shown). For positive candidates, subsequent rounds of PCRs were performed to demonstrate the desired HDR event comparing the homozygous (deletion allele over a deficiency), heterozygous (deletion allele over *CyO*) and wild type (the corresponding injection line) for *RluA-1*^*del-HDR*^, *RluA-2*^*del-HDR*^ and *RluA-1*^*del-HDR*^*RluA-2*^*del-HDR*^ (primer pairs marked in Figures S4A and S6A, PCR amplification in Figures S4B and S6B). PCR products sequenced across the *attp-loxP-3XP3-DsRed-SV40-loxP* fragment (Figure S4C and Figure S6C) confirmed accurate targeting of the loci. For behavioral analysis, the original deletion mutants were backcrossed to *CS*, *w^1118^* or iso*w^1118^* for six times. For each generation, five heterozygous females were selected for six successive backcrosses in vials and about 10 heterozygous females were used to cross to a second chromosome balancer to produce balanced mutant males in bottles and finally heterozygous mutant virgins and balanced males were crossed *en masse* in bottles to establish the balanced and homozygous mutant lines. In all crosses the DsRed fluorescence marker was used to follow the presence of the mutant.

### Generation of deletion line in RluA-1 and RluA-2 using FRT-mediated deficiency

FRT bearing insertions *{WH+}^f02750^* and *{XP-}^d2586^* and in *RluA-1* (locations marked in Figure S4A) were used for generating a deficiency allele *RluA-1*^*del-FRT*^. Insertions of *{WH-}RluA-2*^*F07702*^ and *{WH-}Grip75^f05483^* (locations marked in Figure S6A) were used for generating the deficiency allele *RluA-2*^*del-FRT*^. Crossing and heat-shock schemes followed (Parks *et al.* 2004). Hybrid PCR with corresponding primers (WH5′ plus / XP5′ minus left and right primers) was used to screen for candidate lines with *w*- deletion in *RluA-1*^*del-FRT*^ and two-sided PCR with left and right primers for WH3′ minus/ WH5′ minus was used for screening for candidate lines with *w+* deletion in *RluA-2*^*del-FRT*^* (**Parks et al. 2004**)*. Molecular testing for the deletion was performed by PCR on gDNA extracted from positive candidates placed over a deficiency (*Df(2L)Exelixis7048*) covering the *RluA-1* and *RluA-2* region. The primers used for PCR and sequencing verification in *RluA-1*^*del-FRT*^ are *RluA-1-d2586*-up-F: 5′-AAAAATGCGGTTTGCCCC-3′, located upstream of the *{XP-}d2586* insertion site and *RluA-1-f02750*-down-R: 5′-AAGGGGTAACAAAAGTCCAG-3′, downstream of *{WH+}f02750* insertion site.

### Generation of RluA-1^GAL4^ using “Trojan-exon”

A triplet Trojan exon donor line on the 3^rd^ chromosome *(yw*; *Sp/CyO*; *pC-(lox-attB2-SA-T2A-Gal4-Hsp70)3)* and an *RluA-1* insertion line containing an intronic MiMIC element (*Mi{MIC}RluA-1*^*[MI06897]*^) were used to generate the *RluA-1*^*Gal4*^ driver using a crossing scheme as described (Diao *et al.* 2015). Candidate males who have lost the *y^+^* selection marker associated with *MiMIC* were crossed to *40XUAS-mCD8*::*GFP* line and animals expressing GFP were selected to establish a stable line. The line with the correct linker (phase 0) was confirmed with sequencing of PCR products amplifying the left side with primers *RluA-1*-5494F: 5′-TGATGTTGCCCCATAACG-3′ and *T2A-Gal4*-Seq-1R: 5′ CGCTATCGATGCTCACGGTC-3′ and the right side with the primers *T2A-Gal4*-4F: 5′-ACACCGTGCTGATGCTGC-3′ and *RluA-1*-5907R: 5′-GAAAACATCGCACATCTGG-3′ of the *RluA-1* genome-*T2A-Gal4* insertion bordering region.

### Generation of GFSTF insertions in RluA-1 and RluA-2 by recombination mediated cassette exchange (RMCE)

Crossing, heat shocking and screening for EGFP tagged MiMIC lines in *RluA-1* and *RluA-2* were essentially carried out as described (Nagarkar-Jaiswal *et al.* 2015). Males carrying a MiMIC insertion in a coding intron of *RluA-1* (*RluA-1*^*MI06897*^) or *RluA-2* (*RluA-2*^*MI12981*^) were crossed to females carrying the *hs-FLP* and *vasa-phiC31* integrase on the X chromosome and a frame-specific (“phase 0”) *FRT* flanked multiple tag (*GFSTF*) cassette on chromosome III. Candidate males with mosaic *w*- eyes and *y*- bodies were individually crossed to *w*; *Sco/CyO*; *Sb/TM3 Ser* balancers to establish stocks. The presence and direction of the insertion were tested by PCR assays described (Venken *et al.* 2011). Since the original MiMIC insertion in *RluA-1* or *RluA-2* and the respective gene are in the same orientation, positive PCR reaction 1 (with primers MiLF and TagR) and 4 (with primers MilR and TagF) as described (Venken *et al.* 2011) indicated a successful RMCE event and resulted in expression.

### Generation of UAS-RluA-1

*Drosophila **RluA-1* full length cDNA clone for *RluA-1* transcript A (FI04540) was obtained from the Drosophila Genome Resource Center (DGRC). The *UAS-RluA-1* expression constructs were generated with the ENTR/gateway system following the instructions of the manufacturer (Invitrogen). The *RluA-1*-cDNA-F (5′-CACCATGCAGAATTCTCCGGCT-3′, and *RluA-1*-cDNA^+^STOP-R (5′-TCATGCCGAGTCTAAGTG-3′ primers were used to amplify the open reading frame and cloned into *pENTR-D-TOPO* vector. The sequence was verified and then cloned to the *P*-element based destination vector *pTW*. Model System Injections performed injections into *w^1118^* embryos. F_0_ flies were crossed to *w^1118^* and single F_1_ founders were identified based on the *w*^+^ marker. Individual lines were mapped and balanced to establish stable stocks. A *UAS-RluA-1* line with relatively weak expression evaluated by Q-PCR in *RluA-1*-cDNA lines driven by *md-GAL4* was used for the cIV-neuron specific *RluA-1*-cDNA rescue experiment.

### Larval behavioral analyses

Wandering 3^rd^ instar larvae were washed out from vials and acclimated for 5 min in petri dishes before testing. Larval thermal nociception assays were conducted essentially as previously described (Caldwell and Tracey 2010; Mauthner *et al.* 2014; Walcott *et al.* 2018), except that the probe is gently held against the lateral surface of abdominal segments 4, 5, or 6 until the animal completes a 360° roll along the dorsal-ventral axis. All animals tested eventually performed rolling and the response latency from all the animals was graphed for a given genotype. Larval mechanical nociception response assays were conducted as previously described (Mauthner *et al.* 2014). Behavioral recording and scoring were performed with the observer blinded to the genotype.

For gentle touch assays, early L3 larvae were scooped out from the top layer of the fly food in the vials and 5-10 larvae were briefly rinsed with PBS and allowed to acclimate on 1% agarose in a plate for 5 min before testing with an eyelash fixed to the end of a paintbrush. Each larva was brushed with the eyelash on segments T1-A3 for 4 times and the responses were recorded and summarized using a gentle touch scale (Tsubouchi *et al.* 2012).

### Immunohistochemistry and microscopy

The following primary antibodies were used for immunofluorescence: rabbit anti-GFP (ab6556, Abcam, 1:500), mouse anti-GFP (ab38689, Abcam, 1:500), rabbit anti-HRP (1:100), mouse anti-nc82 (DSHB, supernatant, 1:30). Alexa Fluor 488, 633 were used at 1:1000 as secondary antibodies. Detailed immunostaining protocol is available on request. Images for immunostained tissues were taken on a Zeiss LSM 5 LIVE confocal microscope using a 40X objective except for Figure 2D and Figure S2B, which were taken on a Zeiss LSM880 using the 63X objective.

### CIV dendrite imaging, tracing and analysis

To image class IV dendrites, the DsRed marker in *RluA-1*^*del-HDR*^ was first removed with CRE recombinase (*w^1118^*; *RluA-1*^*del-HDRΔDsRed*^) and *ppk1.9-CD4*::*tdTom* was introduced to the mutant background and a stable homozygous stock was established. For CIV dendrite analysis, six virgins and three males were crossed in each vial for the mutant (*w^1118^*; *RluA-1*^*del-HDRΔDsRed*^; *ppk1.9-CD4*::*tdTom*) and control *(ppk1.9-CD4*::*-td-Tom*). Wandering larvae were anesthetized with diethyl ether in a sealed glass chamber for 15min before being arranged on a slide and covered with 50 mm glass coverslip. Neurons expressing the fluorescently tagged markers were visualized on a Zeiss LSM 5 Live confocal microscope with a 40X oil objective (Plan Apo M27, NA 0.8). Images were collected as 5x3 tile scans of z-stacks with 512x512 resolution. A MatLab build was used for initial automatic tracing of the ddaC neuron dendrites from the confocal z-stack series TIFF images (Gulyanon *et al.* 2016). The generated SWC files were overlaid onto the maximum intensity projected image of the neuron in neuTube (Feng *et al.* 2015) and manually curated to eliminate tracing errors made by MatLab. The corrected images were then analyzed with MatLab to extract neuron features of interest including number of branches, average branch length, and neuron size (the estimated size of the neuron, defined as the area of the minimum bounding circle) (Gulyanon *et al.* 2016). Iso-neuronal crossover events were quantified manually from the traced dendrites for each genotype (n = 6 neurons).

### Statistical analysis

Statistics were performed using GraphPad Prism 4. Thermal and gentle touch behavioral data were compared with an unpaired non-parametric Mann-Whitney test when comparing two groups and Kruskal-Wallis test when comparing three or more groups. Mechanical nociception behavioral data were compared with Fisher’s exact test. Dendrite morphology data were compared with the Student’s *t*-test. Error bars represent standard deviation (S.D.) in all the figures unless otherwise specified. Q-RT-PCR data were analyzed and plotted with Estimation Stats (Ho *et al.* 2019).

### Data availability

Strains and plasmids are available upon request. All supplementary figures have been uploaded to figshare. The authors affirm that all data necessary for confirming the conclusions of the article are present within the article and its figures. Supplemental material available at figshare: https://doi.org/10.25387/g3.12755873.

## Results

### Structural Conservation of RluA-1 and RluA-2 With Pseudouridine Synthases

**Figure 1:**
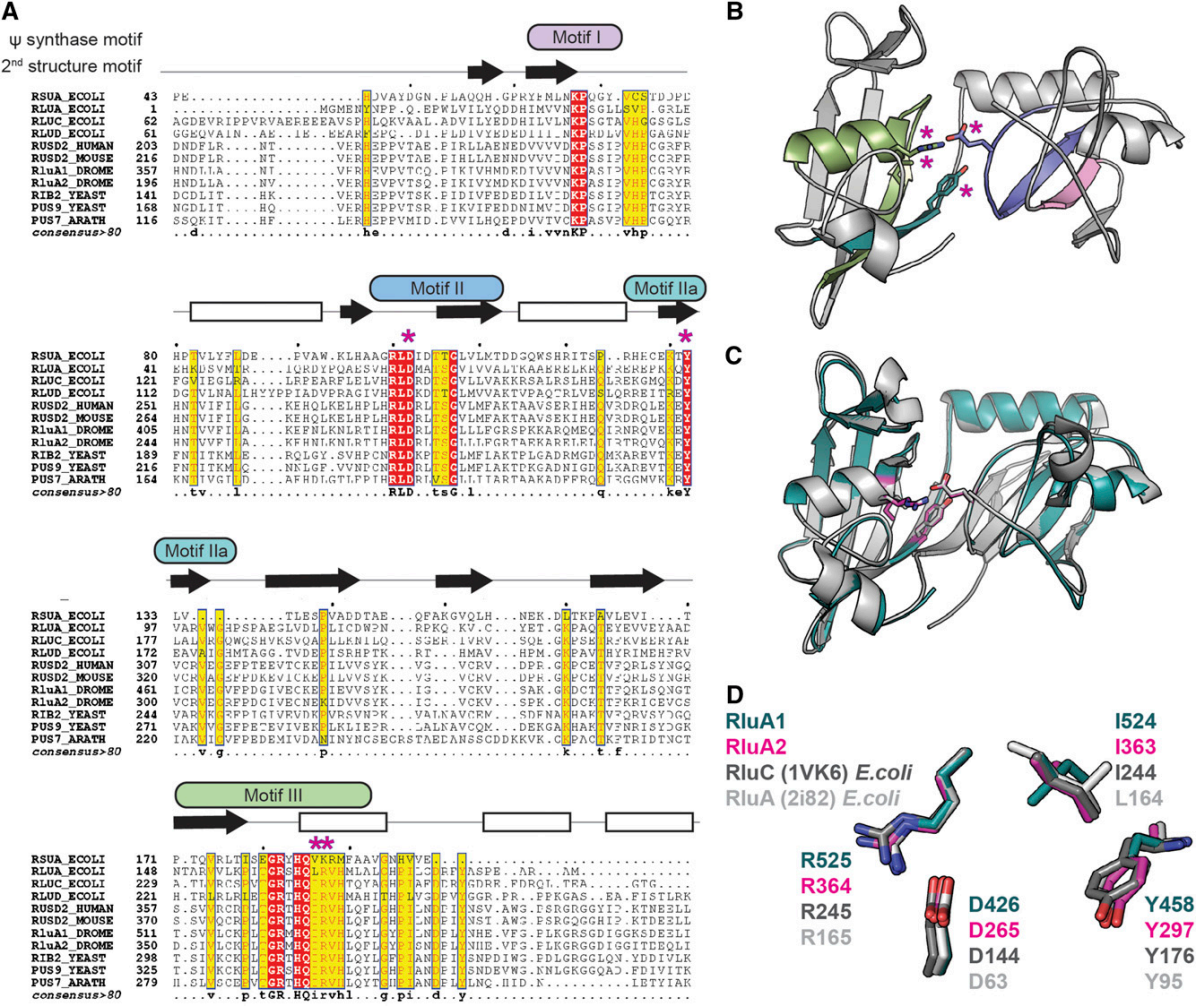
RluA-1 and RluA-2 show significant sequence and structural similarity with pseudouridine synthases. (A). Sequence alignment of RluA-1 and RluA-2 with pseudouridine synthase proteins in different organisms. Alignment includes structural information of *E. coli* RluA (PDB ID 2i82). Positions of the classical pseudouridine synthase motifs I, II, IIa, and III are indicated by colored boxes. Conserved active site residues are indicated with magenta asterisks above sequence. Residues of 100% conservation are boxed in red, residues down to 80% conservation are boxed yellow. Predicted secondary structure elements are indicated above the sequence, β-sheets as black filled arrows pointing right (N- to C-terminus) and ⍺-helices as open rectangle. (B). Structural homology model of RluA-1 showing location of Ψ synthase motifs I, II, IIa, and III and conserved residues Asp (D) 426, Tyr (Y) 458, Ile (I) 524 and Arg (R) 525 indicated with magenta asterisks. (C). Superimposition of RluA-1 model in teal color and *E. coli* RluA in gray. (D). Superimposition of conserved Ψ synthase residues DYI/LR of models RluA-1 (teal), RluA-2 (pink), *E. coli* RluC (dark gray) and *E. coli* RluA (light gray).

Both RluA-1 and RluA-2 are annotated as putative pseudouridine synthases but their Psi synthase activities have not yet been proven biochemically. Thus, we explored features of their predicted amino acid sequences to further scrutinize their hypothesized enzymatic activity. First, we aligned the predicted amino acid sequences of RluA-1 and RluA-2 with their closest homologous pseudouridine synthases across the kingdoms of life. This sequence alignment shows that RluA-1 and RluA-2 are highly conserved throughout the entire predicted pseudouridine synthase domain (Figure 1A). Importantly, highly conserved residues of pseudouridine synthase domain are distributed across the primary amino acid structure in four sequence motifs (motif I, motif IIa, motif IIb and motif III) as defined by Koonin (Koonin 1996). These motifs harbor key residues that are close to 100% conserved across superfamily members and species. Each of the fully conserved non-catalytic residues (*i.e.*, motif I, Figure 1A) and critical residues thought to be important for catalysis (asterisks, Figure 1A) are conserved in both RluA-1 and RluA-2. To further evaluate the level of structural conservation within the pseudouridine synthase domains of RluA-1 and RluA-2, we generated structural homology models based on solved structures of pseudouridine synthases. Interestingly, we found that the best model for RluA-1 was based on *E. coli* RluA (PDB ID 2i82), whereas the best RluA-2 model was generated from the template structure of *E. coli* RluC (PDB ID 1vk6) (Figure 1B, 1C, Figure S1A, S1B). In our model, key residues and their side chains (DYI/LR), within motifs II, IIa and III of RluA-1 and RluA-2 models, are clearly superimposed with the position of the template residues in 3D space (Figure 1D). Together, the primary sequence and homology model analyses provide strong evidence of conservation of function of pseudouridine synthase domains in RluA-1 and RluA-2, supporting the hypothesis that RluA-1 and RluA-2 act as pseudouridine synthases, leaving to future investigation the nature of their substrates and whether they interact with RNA similarly as other synthases (Figure S1C, S1D). However, we cannot exclude the possibility that these enzymes may possess additional activities. For instance, both RluA-1 and RluA-2 possess amino acid similarity to an RNA-binding S4 domain (located N-terminal to the Psi synthase domain (data not shown)).

### RluA-1 is expressed in the multidendritic neurons of the peripheral nervous system and in the cells of brain

**Figure 2:**
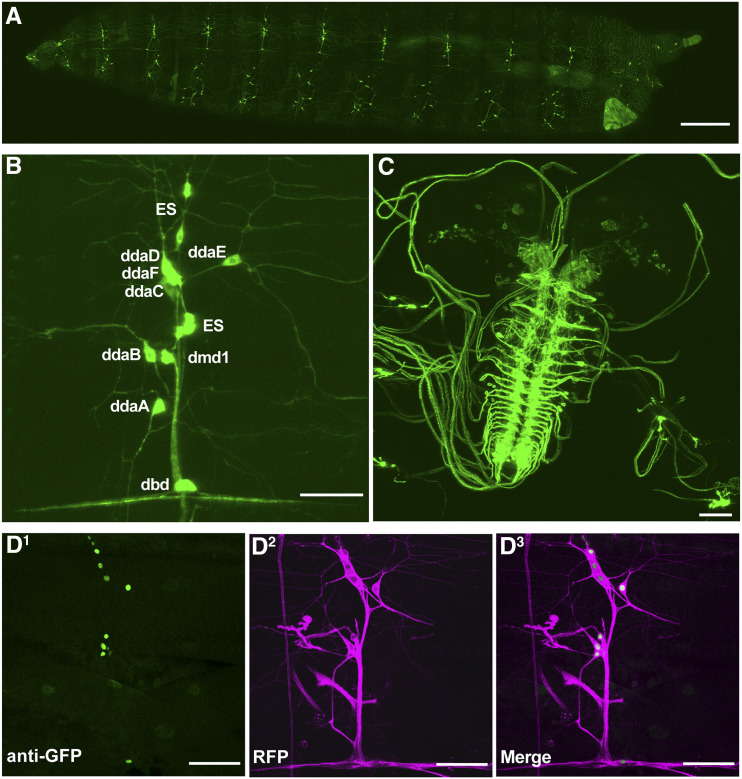
*RluA-1* gene and protein expression in *Drosophila melanogaster*. (A) A low magnification confocal micrograph of *RluA-1* gene expression pattern in the larval PNS (third instar *w^1118^*; *RluA-1*^*Gal4*^/*40xUAS-mCD8*::*GFP*). Note that GFP positive signals are detected in peripheral sensory neurons in each segment along the larval body wall. Scale bar = 500μm. (B) Higher magnification of an abdominal dorsal PNS cluster. *RluA-1*^*Gal4*^ is expressed in the cell body and dendrites of all classes of multidendritic neurons, external sensory (ES), dorsal multiple dendrite neuron (dmd1) and dorsal bipolar dendritic (dbd) neurons (ddaD and ddaE (Class I), ddaB (Class II), ddaA and ddaF (Class III), and ddaC (ClassIV), ES, dmd1 and dbd neurons are labeled). Scale bar = 50μm. (C). *RluA-1*^*Gal4*^*/+* driving expression of *40xUAS-mCD8*::*GFP/+* in the larval CNS. Labeling is observed in axonal projections of sensory neurons in the larval ventral nerve cord and unidentified clusters of neurons in the larval brain. Scale bar = 50 μm. (D^1-3^) anti-GFP immunohistochemistry of a third instar larval fillet preparation of *RluA-1-GFSTF/ Gal4109(2)80*>*UAS-mCD8-RFP*, immunoreactive signals are detected in the nuclei of multi-dendritic neurons (D^1^ green, GFP) surrounded by the membrane-localized RFP signal (D^2^ magenta, RFP) and merged image in D^3^. Scale bar = 50μm.

Previously described reporter genes for *RluA-1* showed specific expression in larval multidendritic neurons (Wang *et al.* 2011). We replicated this finding by generating an *RluA-1*^*GAL4*^ driver at the endogenous gene locus through recombination mediated cassette exchange (RMCE) of *RluA-1*^*MI06897*^ (an intronic MiMIC) and a Trojan exon cassette (Diao *et al.* 2015). An mCD8GFP reporter driven by *RluA-1*^*GAL4*^ was expressed in peripheral sensory neurons in each segment of the larval body wall (Figure 2A). In the dorsal cluster, GFP-positive signals were clearly detected in all four classes of md-da sensory neurons, dorsal multiple dendrite neuron (dmd1), external sensory (ES) and dorsal bipolar dendritic (dbd) neurons (Figure 2B). In the larval ventral nerve cord, the GFP-positive signals were seen in the axonal projections of the sensory neurons (Figure 2C). GFP signal was also observed in the unidentified clusters of neurons in the larval brain (Figure 2C). In the adult brain, significant signals were detected in the optic lobes and other small cell clusters of the central brain (Figure S2A).

To determine the localization of the RluA-1 proteins, we generated a GFSTF exon trap (Nagarkar-Jaiswal *et al.* 2015) that expresses an in frame GFP fusion with *RluA-1* at the endogenous genomic locus (with RMCE of the *RluA-1*^*MI06897*^ MiMIC element) (Nagarkar-Jaiswal *et al.* 2015). Although live imaging did not detect the EGFP tagged RluA-1 protein, immunostaining with anti-GFP labeled nuclei of larval multidendritic neurons, ES and dbd neurons also expressing a membrane-localized RFP (*Gal4109(2)80* > *UAS-CD8-RFP*, Figure 2D). The tagged RluA-1 protein was also detected in the cell bodies of neurons in optic lobes and other yet-to-be identified cells in the adult brain (Figure S2B).

### Reducing or removing RluA-1 results in a hypersensitive thermal nociception phenotype

**Figure 3:**
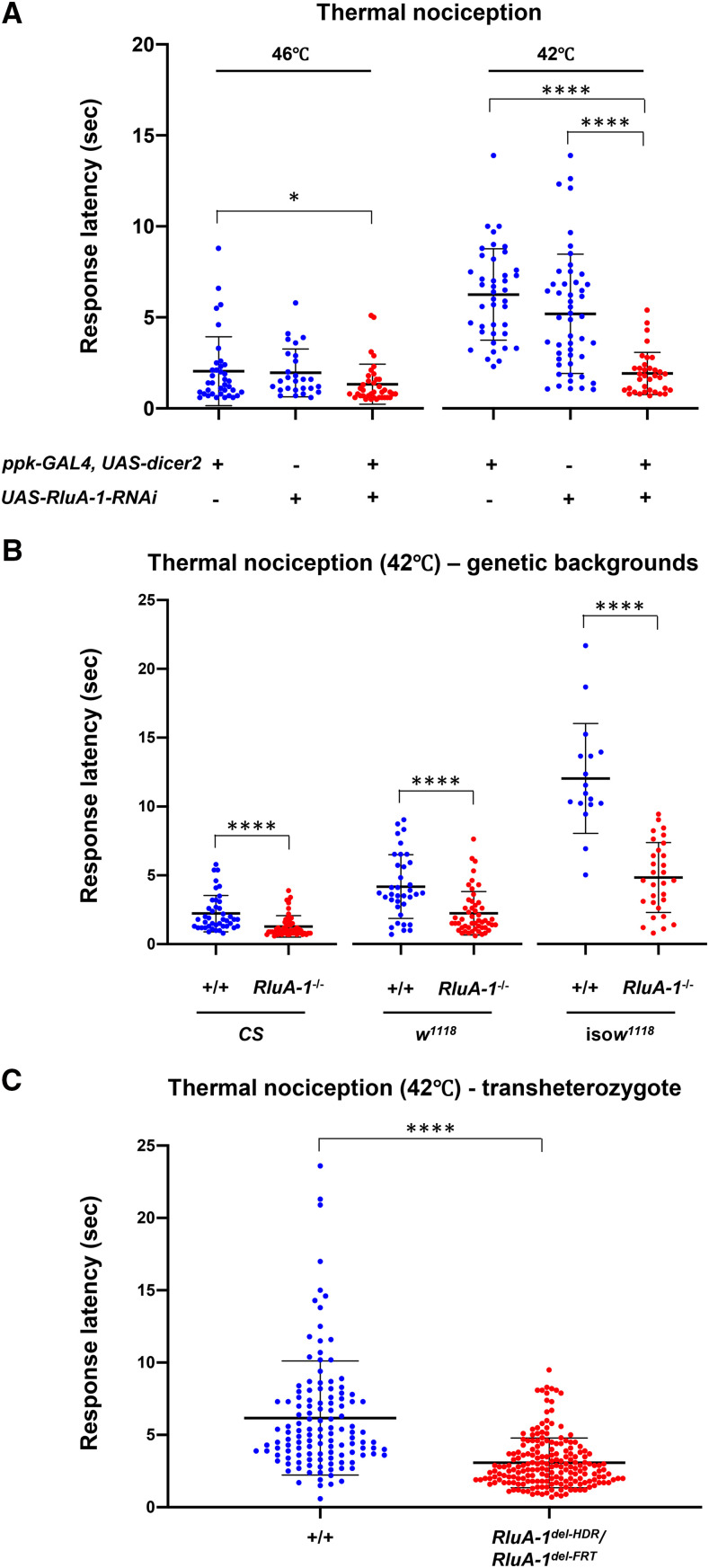
Thermal nociception in animals with *RluA-1* loss of function. (A). Class IV specific knock-down in *RluA-1* results in significant hypersensitive thermal nociception in larvae compared to the *ppk-GAL4* driver alone animals at 46° (average latency of 1.33 ± 1.10 sec for *ppk-Gal4* > *UAS-RluA-1-RNAi (*31719-R1*)*, n = 38 *vs.* 2.04 ± 1.89 sec for *ppk-GAL4* driver alone, n = 37. *P* < 0.05), albeit not statistically significant compared to the *UAS-RluA-1RNAi* alone (1.96 ± 1.32 sec for *UAS-RluA-1RNAi* alone, n = 27). These differences are more pronounced at 42° (average latency of 1.92 ± 1.16 sec for *ppk-Gal4* > *UAS-RluA-1-RNAi*, n = 35 *vs.* 6.25 ± 2.51 sec for *ppk-GAL4* driver alone controls, n = 42 or 5.20 ± 3.28 sec for *UAS-RluA-1RNAi* alone controls, n = 49, *P* < 0.0001). Kruskal-Wallis test with Dunn’s multiple comparison tests, only significant comparisons were labeled. (B). Homozygous null mutant *RluA-1*^*del-HDR*^ larvae showed significantly faster response to noxious heat stimulation of 42° compared to the corresponding control animals of *Canton-S* (average latency of 1.29 ± 0.78 sec in *RluA-1*^*−/−*^, n = 54 *vs.* 2.22 ± 1.32 sec in *CS*, n = 39), *w^1118^* (2.23 ± 1.57 sec in *RluA-1*^*−/−*^, n = 51 *vs.* 4.16 ± 2.31 sec in *w^1118^*, n = 36) and iso*w^1118^* (4.82 ± 2.53 sec in *RluA-1*^*−/−*^, n = 30 *vs.* 11.99 ± 3.98 sec in iso*w^1118^*, n = 17). Significance of comparisons are marked as **** (*P* < 0.0001). Data were analyzed using Mann-Whitney non-parametric test. (C). Transheterozygote *RluA-1*^*del-HDR*^/*RluA-1*^*del-FRT*^ showed hypersensitive thermal nociception responses compared to the controls (average latency of 3.07 ± 1.72 sec in *RluA-1*^*del-HDR*^*/RluA-1^del-FRT^*, n = 195 *vs.* 6.17 ± 3.95 sec in +/+, n = 125). The genetic background is *w^1118^* for *RluA-1*^*del-HDR*^, and iso*w^1118^* for *RluA-1*^*del-FRT*^. For the *RluA-1*^*del-HDR*^*/RluA-1^del-FRT^* transheterozygotes, data were pooled from the progeny from reciprocal crosses of *RluA-1*^*del-HDR*^ to *RluA-1*^*del-FRT*^. To generate the control larvae, reciprocal crosses were made between the genetic background of *w^1118^* and iso*w^1118^* and the data from the progeny of these crosses were pooled. Significance of the comparison is marked as **** (exact *P* < 0.0001). Data were analyzed using Mann-Whitney *U*-test. Error bars in all the figures represent S.D.

Given our confirmation of the expression of the *RluA1* gene and protein in the multidendritic neurons we tested for potential roles of RluA-1 in the regulation of nociception. To do so, we first performed tissue-specific knockdown using GAL4/UAS based RNA interference (RNAi) (with a UAS line that showed a trend in reducing the expression of *RluA-1* transcripts in multidendritic neurons (Figure S3)). To investigate the behavioral consequence, a cIVda specific driver (*ppk1.9-GAL4*; *UAS-dicer2*) (Ainsley *et al.* 2003) was employed to drive the *RluA-1-RNAi* in the larval cIVda nociceptors. We assessed potential insensitive phenotypes (with a probe temperature of 46°) and potential hypersensitive phenotypes (with a temperature of 42°) as previously described (Honjo *et al.* 2016). When stimulated with the higher temperature 46° probe the *RluA-1-RNAi* knockdown larvae responded significantly faster than the *ppk-GAL4* driver alone controls. This genotype also showed a trend toward responding faster than the no driver *UAS-RNAi/+* controls but this difference was not statistically significant (Figure 3A). The results suggested that reducing *RluA-1* in classIV neurons may have made the larvae more sensitive to noxious heat. Indeed, this was clearly observed for the *RluA1*-RNAi animals when testing with 42° probe that allows for easier detection of hypersensitivity (Figure 3A). The average latency to roll in the *RluA-1* knock-down animals was significantly faster than the driver alone animals or the *UAS-RNAi* control animals. These data combined suggest that reducing the activity of *RluA-1* in the noxious heat-responsive clVda cells caused thermal hyperalgesia.

To further test the function of *RluA-1* we next generated a precise genetic deletion mutant of *RluA-1* in which 11.14 kbp including the entire *RluA-1* genomic region was removed by CRISPR-guided homologous recombination-directed repair (HDR) (Ran *et al.* 2013). Homology arms of ∼1kb immediately flanking the CRIPSPR cleavage sites were used to direct the HDR (Figure S4A). The resultant deletion mutant (*RluA-1*^*del-HDR*^) was confirmed by PCR amplification and sequencing of PCR products from the targeted *RluA-1* locus (Figure S4B and S4C). To facilitate behavioral comparisons, the *RluA-1*^*del-HDR*^ mutant was backcrossed six times to commonly used strains Canton-S (CS), *w^1118^*, and isogenized *w^1118^* (iso*w^1118^*). In all of the tested genetic backgrounds *RluA-1*^*del-HDR*^ larvae showed significantly faster responses to noxious heat stimulation of 42° compared to the corresponding control strain animals (Figure 3B). Note that these dissimilar genetic backgrounds (Canton-S was originally collected in Canton Ohio in 1968, *w^1118^* is derived from Oregon-R collected in the wild prior to 1928 while iso*w^1118^* was a highly inbred version of *w^1118^* (Thurmond *et al.* 2019)) vary in their baseline responses. Nevertheless, homozygous *RluA-1*^*−/−*^ mutants showed hypersensitive nociception phenotypes regardless of background. The most striking differences were between homozygous *RluA-1*^*−/−*^ and the relatively insensitive iso*w^1118^* background, followed by that of *w^1118^* background, and then the CS background (Figure 3B).

We performed an additional genetic test for the importance of *RluA-1*, by generating an independent mutant allele (*RluA-1*^*del-FRT*^) using FLP recombinase and FRT-bearing insertions (Parks *et al.* 2004) (*PBac{WH}^f02750^*(+) and *p{XP}^d2586^*(-), as labeled in Figure S4A). Transheterozygous *RluA-1*^*del-FRT*^/*RluA-1*^*del-HDR*^ mutant larvae displayed hypersensitivity to a 42° stimulus (Figure 3C) indicating that *RluA-1*^*del-FRT*^ failed to complement *RluA-1*^*del-HDR*^. Failure of complementation of independently generated alleles created in distinct genetic backgrounds provides additional strong evidence that the hypersensitive nociceptive phenotypes observed are a consequence of the mutation of *RluA-1*.

For the remainder of our behavioral studies, we focused on the isogenized *w^1118^* background. This had the advantage of greater genetic uniformity relative to *w^1118^* and CS, as well as showing the strongest hypersensitive mutant phenotype for *RluA-1*.

### Genetic rescue of RluA-1 mutant restores thermal nociception response

**Figure 4:**
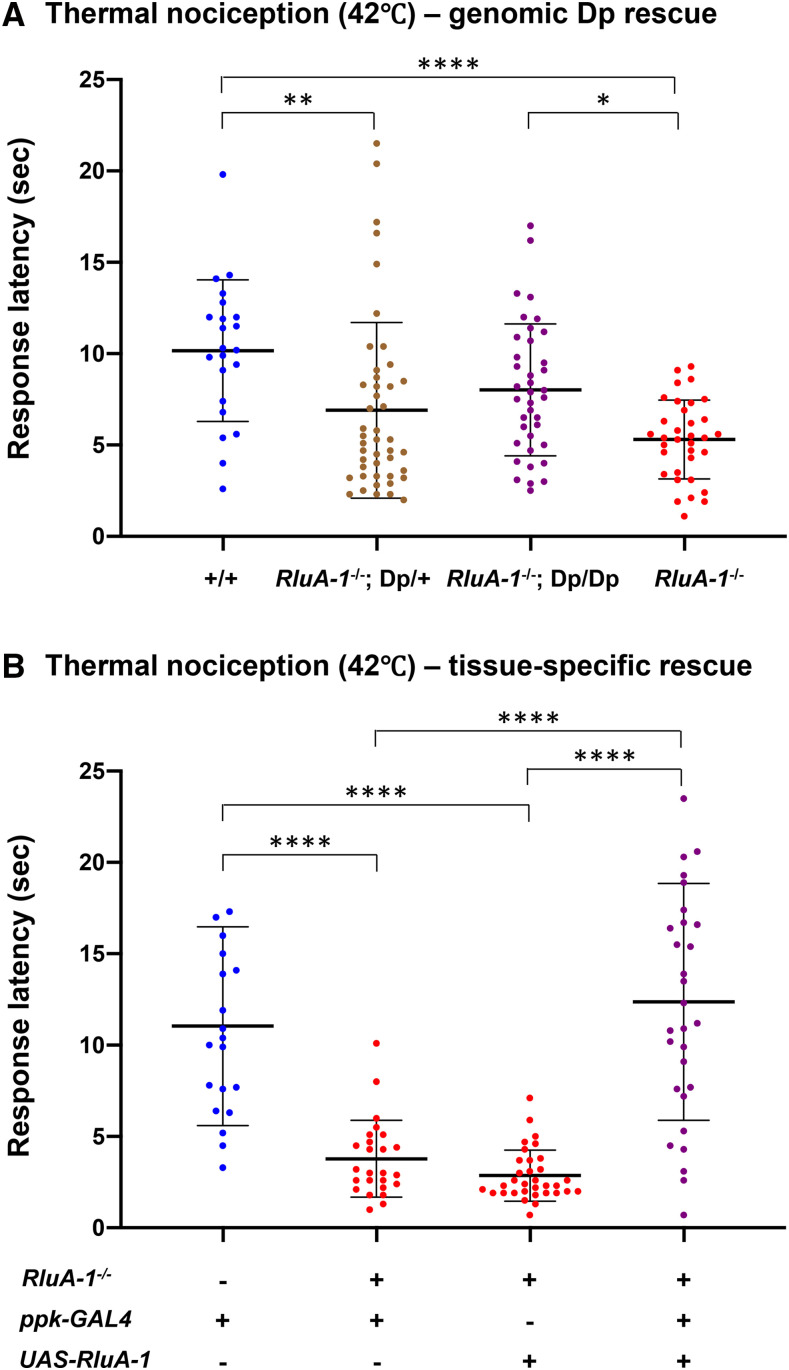
*RluA-1* nociception phenotypes with (A) chromosome duplication or (B) Class IV-specific expression of *UAS-RluA-1 cDNA*. (A). The hypersensitive nociception phenotype with 42° thermal stimulus in *RluA-1*^*del-HDR*^ null mutant was rescued by introducing a duplication (Dp) on the third chromosome which covers the *RluA-1* gene region (average latency of 8.02 ± 3.61 sec in *RluA-1*^*−/−*^; Dp/Dp, n = 38, which is similar to the iso*w^1118^* genetic background (+/+, average latency of 10.16 ± 3.87 sec, n = 22), but significantly slower than the latency of 5.31 ± 2.16 sec in *RluA-1*^*−/−*^, n = 34 (*P* = 0.02)). Larvae with one copy of duplication showed slower response (average latency of 6.90 ± 4.81 sec in *RluA-1*^*−/−*^; *Dp/+*, n = 45) without statistical significance compared to *RluA-1*^*del-HDR*^. Data were analyzed using Kruskal-Wallis test with Dunn’s multiple comparison tests. (B). The hypersensitive thermal nociception phenotype in *RluA-1*^*del-HDR*^ larvae was completely reversed to that of the heterozygous *RluA-1* by Class IV specific expression of full length *RluA-1-cDNA* (*RluA-1*^*−/−*^; *ppk-Gal4^+/−^*; *UAS-RluA-1*^*+/−*^, average latency of 12.37 ±6.48 sec, n = 30, is similar to *RluA-1*^*+/−*^; *ppk1.9-Gal4^+/−^*, average latency of 11.04 ± 5.44 sec, n = 20 but significantly slower than either the driver alone (*RluA-1*^*−/−*^; *Ppk1.9-Gal4*^+/−^, average latency of 3.78 ± 2.11 sec, n = 25 or transgene alone controls (*RluA-1*^*−/−*^; *UAS-RluA-1*^+/−^, average latency of 2.86 ± 1.39 sec, n = 33) at 42°C thermal stimulus. Significance of the comparisons are marked as **** (*P* < 0.0001). Data were analyzed using Kruskal-Wallis test with Dunn’s multiple comparison tests. Error bars in all the figures represent S.D.

To test that the mutation in *RluA-1* was the underlying cause of the hypersensitive nociception phenotype, we introduced a genomic rescue construct into the *RluA-1*^*del-HDR*^ mutant background (a Bacterial Artificial Chromosome (BAC) (P6-D7)) (Venken *et al.* 2009) covering the *RluA-1* gene region). With the 42° stimulus, larvae with two copies of the duplication (Dp) in the background of *RluA-1*^*del-HDR*^ showed rescue of the hypersensitivity (Figure 4A). This rescue with the genomic duplication was dosage dependent. Larvae with only one copy of the Dp in the *RluA-1*^*del-HDR*^ background (*RluA-1*^*−/−*^; *Dp*^*+/−*^) responded more slowly than the mutant but this difference was not statistically significant (Figure 4A). Combined, the results of genomic rescue experiments support the hypothesis that mutation of *RluA-1* is indeed the cause of the hypersensitive thermal nociception phenotype.

A caveat remained in that the genomic rescue construct included other genes in addition to *RluA-1*. Thus, genomic rescue did not rule out the possibility that a mutation tightly linked to *RluA-1*, but not in *RluA-1* itself, was responsible for the mutant phenotype. Thus, as a final test, we generated transgenic lines to express an *RluA-1* cDNA under the control of the GAL4/UAS system (*UAS-RluA-1*). Using the *UAS-RluA-1* we specifically restored *RluA-1* to cIVda neurons in the *RluA-1* null mutant background. When stimulated with the 42° probe the animals with both the *ppkGal4* driver and the *UAS-RluA-1-cDNA* transgene in the *RluA-1^del-HDR^* showed a complete rescue from the hypersensitivity seen in the null mutant (Figure 4B). Neither the *ppkGal4* driver alone nor the *UAS-RluA-1* had an effect on the hypersensitive thermal nociception phenotype in the *RluA-1* null mutant background, excluding the possibility of non-specific effects of these transgenes (Figure 4B). In addition, overexpression of *UAS-RluA1* with a md neuron driver (MD-Gal4) had no effect on nociception behavior at 42° stimulus ruling out the possibility that the increased latency seen in the rescue effect was a non-specific consequence of over-expression (Figure S5). Combined, these nociceptor-specific rescue experiments provide genetic confirmation that loss-of-function mutation in *RluA-1* causes hypersensitive thermal nociception and localizes the site of action for RluA-1 in this process to the nociceptors.

### RluA-1 requirement for mechanosensory thresholds

**Figure 5:**
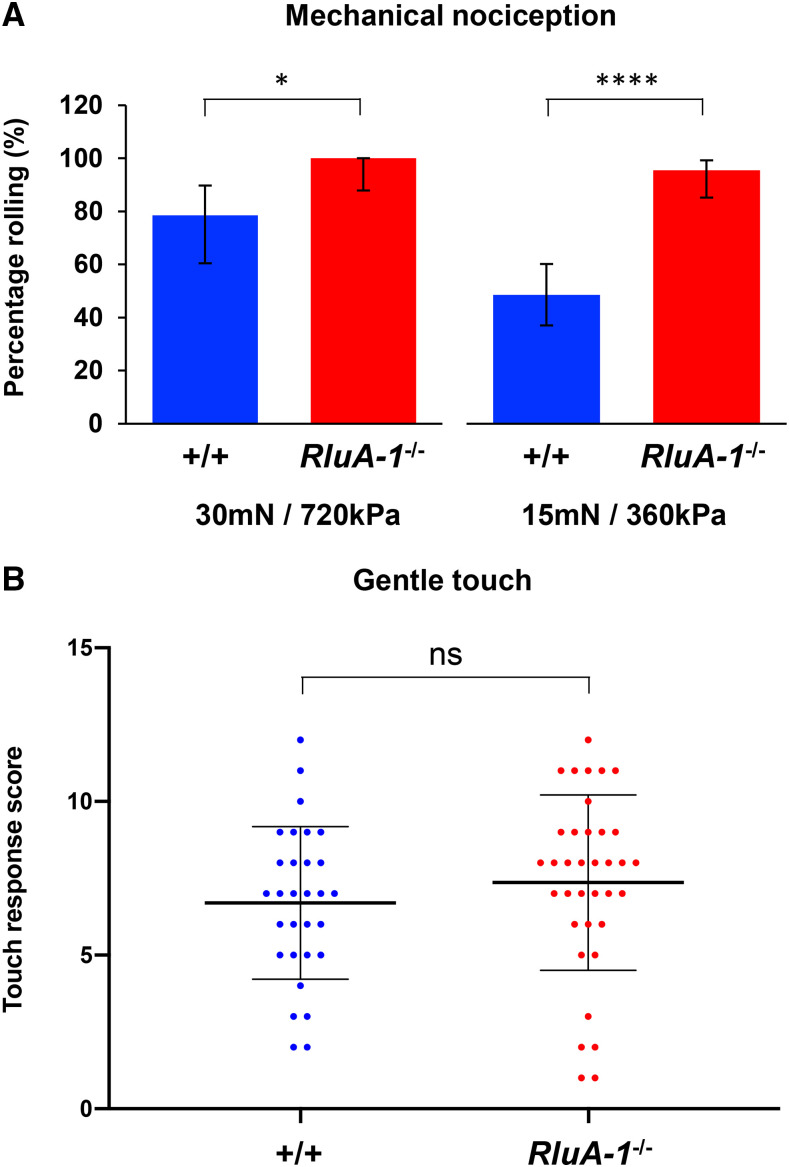
*RluA-1* mechanical nociception and gentle touch responses. (A). In response to the noxious mechanical stimulus of 30mN/720kPa, all (100%) of the *RluA-1*^*del-HDR*^ null mutant larvae (*RluA-1*^*−/−*^, n = 28) rolled compared to the 78.57% of control larvae (iso*w^1118^*, n = 28) rolling. At the reduced stimulus of 15mN/360kPa, 95.56% of *RluA-1*^−/−^ (n = 45) rolled while only 48.53% of control (n = 68) rolled. Significance of the comparisons are marked as *(*P* < 0.05) and **** (*P* < 0.0001). Data were analyzed using Fisher’s exact test and presented as percentages ± 95% confidence intervals. (B). *RluA-1*^*del-HDR*^ larvae (*RluA-1*^−/−^) had a gentle touch response score (6.70 ± 2.48, n = 36) similar to the control larvae (iso*w^1118^*, 7.83 ± 2.69, n = 30). ns, not significant (*P* > 0.05). Data were analyzed with Student’s *t*-test. Error bars represent S.D.

The cIVda neurons are not only required for detection of noxious heat, they also contribute to sensing harsh mechanical stimulation (Hwang *et al.* 2007; Zhong *et al.* 2010; Mauthner *et al.* 2014). Thus, we investigated the *RluA-1* mutant responses to noxious mechanical stimuli. When stimulated with a 30mN/720kPa Von Frey fiber, significantly more *RluA-^del-HDR^* null mutant larvae performed the typical nociceptive rolling behavior compared to the iso*w^1118^* controls (Figure 5A). Even when the probe was reduced to 15mN/360kPa, the majority of *RluA-1*^*−/−*^ larvae still rolled while less than half of control larvae rolled (Figure 5A), indicating the defect in *RluA-1* also caused hypersensitive mechanical nociception. Loss of *RluA-1* did not have any impact on behavioral responses to gentle touch (Figure 5B) suggesting a more specific involvement in nociception than for sensory processing in general.

### Expression pattern and nociception functions for RluA-2

**Figure 6:**
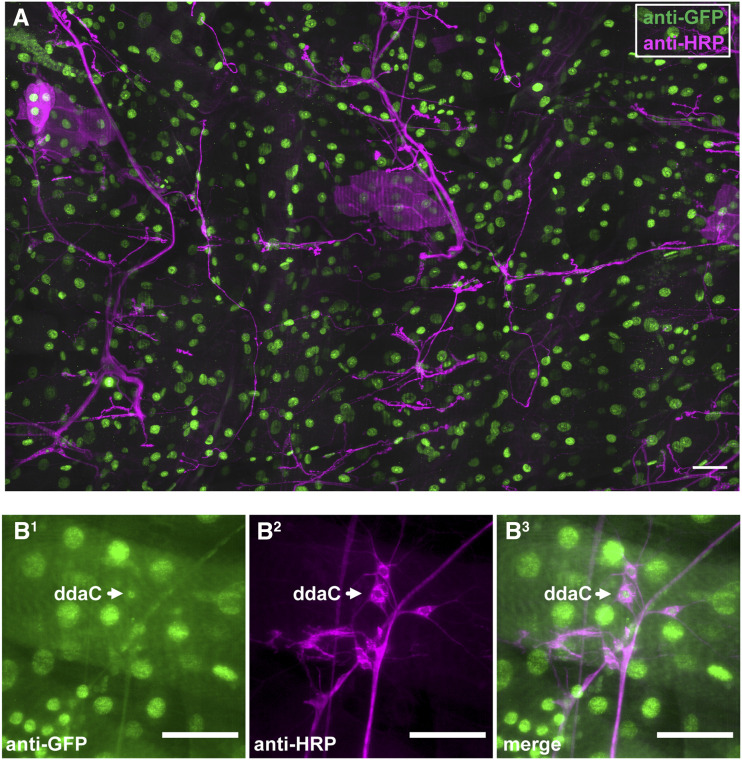
RluA-2 expression in *Drosophila* larvae. (A). In third instar larvae of *RluA-2-GFSTF* line, anti-GFP (green) immunoreactive signals are detected in the nuclei of all types of cells (neurons are labeled with anti-HRP (red)). Scale bar = 100μm. (B). In the larval abdominal dorsal PNS cluster of *RluA-2-GFSTF* line, GFP signal (green) is also detected in the nuclei of the md neurons, whose membranes are marked with anti-HRP (magenta). Arrow points to Class IV ddaC neuron. Scale bar = 50μm.

The *RluA-2* locus is adjacent to *RluA-1* on the second chromosome of *Drosophila melanogaster*. RluA-2 has significant sequence similarity with RluA-1 within the evolutionarily conserved pseudouridine synthase domain (Figure 1, Figure S1), suggesting possible functional overlap for the encoded proteins. To investigate the expression of RluA-2 we generated a GFSTF line with RMCE of the *RluA-2*^*MI12981*^ mimic element (Figure S6A) (Nagarkar-Jaiswal *et al.* 2015). Immunostaining with anti-GFP labeled nuclei in all of the cell types that we observed in third instar larvae (Figure 6A), including md neurons (Figure 6B).

To test the function of *RluA-2* we generated a deletion (*RluA-2*^*del-HDR*^) to remove its pseudouridine synthase domain via CRISPR/Cas9 HDR (Figure S6A-C). Note that *RluA-2*^*del-HDR*^ is not an RNA null allele and some residual function from the RNA binding S4 domain may remain. However, the RluA-2 pseudouridine synthase domain is completely removed. Since RluA-1 and RluA-2 are both expressed in the md neurons (Figure 2D and Figure 6B), we generated a double mutant (*RluA-1*^*del-HDR*^*RluA-2*^*del-HDR*^), by injecting the *RluA-2*^*del-HDR*^ constructs in the *RluA-1*^*del-HDR*^ null mutant background (Figure S6). As RluA-1 and RluA-2 are the only annotated RluA family members in *D. melanogaster*, the double mutant completely removes RluA family pseudouridine synthase activity from the flies. Prior to functional assessment, the single mutant and the double mutant were backcrossed six times to the genetic background of iso*w^1118^*.

**Figure 7:**
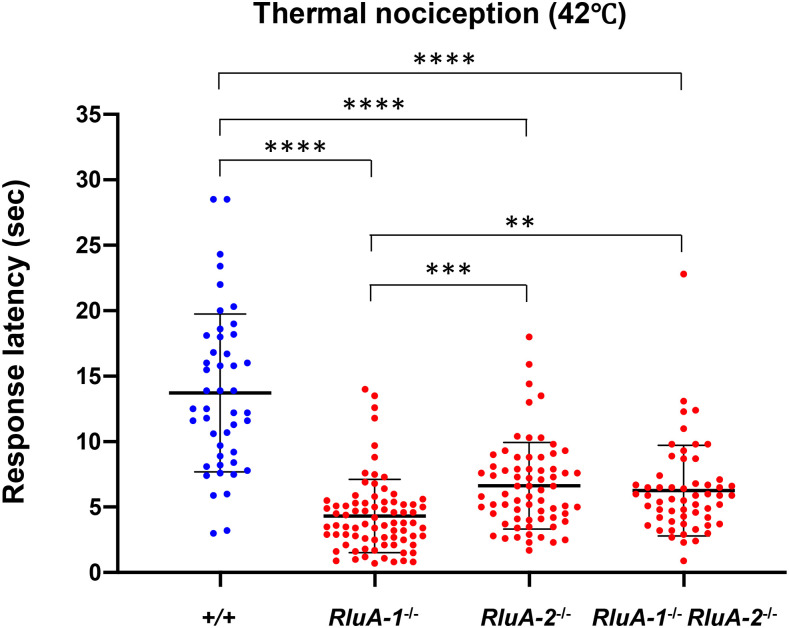
Thermal nociception responses of *RluA-1* and *RluA-2* single mutant and *RluA-1* *RluA-2* double mutant. Larvae of single mutant *RluA-1*^*del-HDR*^ (*RluA-1*^*−/−*^), *RluA-2*^*del-HDR*^ (*RluA-2*^*−/−*^), double mutant *RluA-1*^*del-HDR*^ *RluA-2*^*del-HDR*^ (*RluA-1*^*−/−*^ *RluA-2*^*−/−*^) and the genetic background iso*w^1118^* (+/+) were stimulated with noxious heat probe of 42°. *RluA-1*^*−/−*^, *RluA-2*^*−/−*^, and *RluA-1*^*−/−*^ *RluA-2*^*−/−*^ all displayed faster responses to the stimulus compared to controls (*RluA-1*^*−/−*^ average latency of 4.32 ± 2.80 sec, n= 79; *RluA-2*^*−/−*^, 6.63 ± 3.31 sec, n = 68; (*RluA-1*^*−/−*^*RluA-2*^*−/−*^), 6.26 ± 3.46 sec, n = 57; +/+, 13.72 ± 6.03 sec, n = 46). Significance of comparisons are marked as ** (*P* < 0.01), *** (*P* < 0.001) or **** (*P* < 0.0001). Data were analyzed using Kruskal-Wallis test with Dunn’s multiple comparison tests. Error bars in all the figures represent S.D.

We tested each single mutant (*RluA-1*^*del-HDR*^ and *RluA-2*^*del-HDR*^) together with the double mutant *RluA-1*^*del-HDR*^ *RluA-2*^*del-HDR*^ side by side in thermal nociception assays with the 42° thermal stimulus. *RluA-1*^*del-HDR*^ larvae (*RluA-1*^*−/−*^) again responded significantly faster than the genetic background control (Figure 7). The *RluA-2*^*del-HDR*^ single mutant larvae also displayed a faster response to the stimulus (Figure 7) and similar hypersensitivity was also seen in an independent allele for *RluA-2* that we generated by FLP/FRT mediated recombination (*RluA-2*^*del-FRT*^) allele (Figure S7). Finally, the double mutant *RluA-1*^*del-HDR*^ *RluA-2*^*del-HDR*^ larvae showed a faster response than the control larvae to the same extent as the single mutant of *RluA-2*^*del-HDR*^ (Figure 7). These results indicated that *RluA-2*, like *RluA-1*, negatively regulates nociception. The finding that the double mutant did not show a more severe phenotype than either single mutant suggests that RluA-2 and RluA-1 have non-redundant functional roles, and that they may function in the same molecular pathway. When this pathway is disrupted, hypersensitive nociception results.

### RluA-1 regulates neuronal dendrite morphology of nociceptors

**Figure 8:**
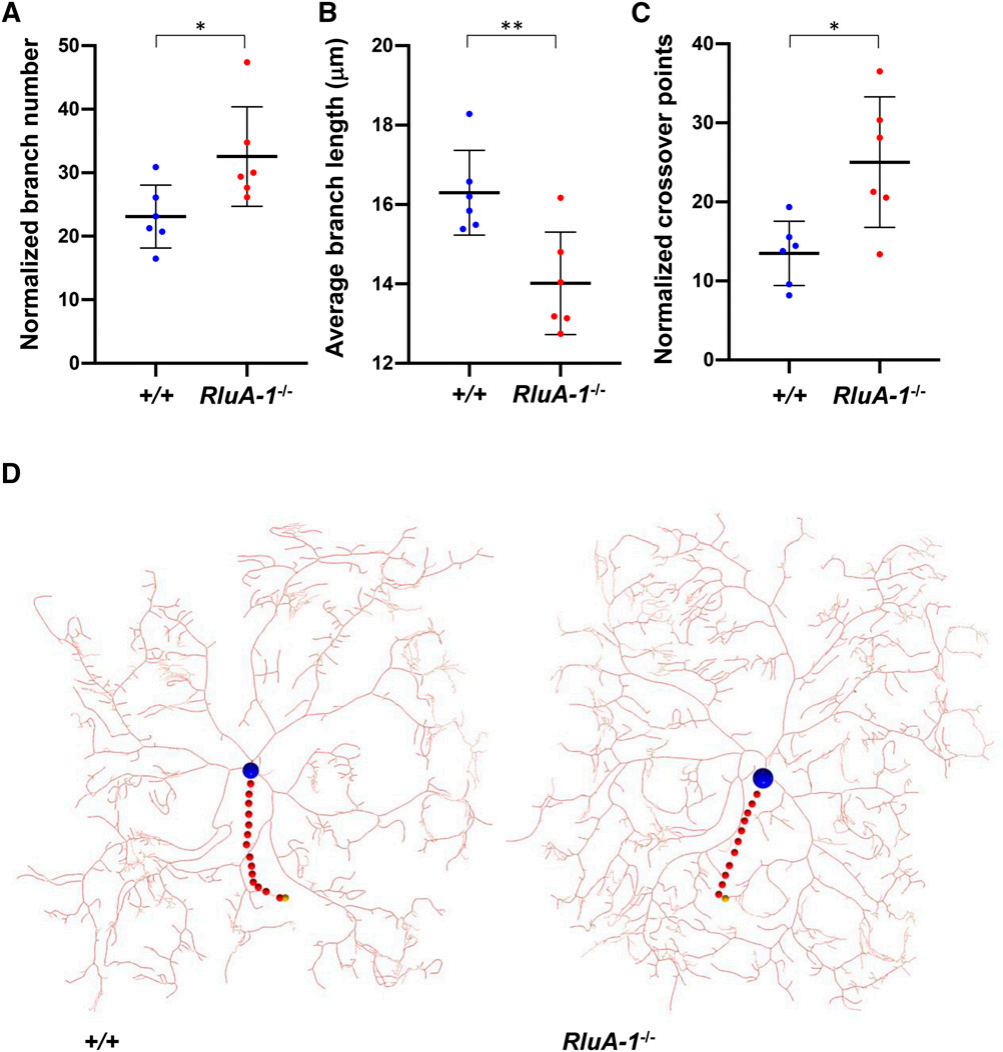
*RluA-1* ddaC neuron dendrite morphology. Quantification of the branch number (A), average branch length (B), and isoneuronal cross-over points (C). (A). Homozygous *RluA-1*^*del-HDR*^ larvae showed a higher normalized branch number (total number of branches / (neuron size × 10^−4^μm^2^), 32.56 ± 7.83 in *RluA-1*^*−/−*^; *ppk-tdTom*, n = 6 *vs.* 23.10 ± 4.96 in *ppk-tdTom* control, n = 6, *P* < 0.05). (B). The mean ddaC dendritic branch length in homozygous *RluA-1*^*del-HDR*^ was shorter than that of the control (average branch length is 14.01 ± 1.29 μm in *RluA-1*^*−/−*^; *ppk-tdTom*, n = 6 *vs.* 16.30 ± 1.07 μm in *ppk-tdTom* control, n = 6, *P* < 0.01). (C). The number of ddaC dendritic isoneuronal crossovers in homozygous *RluA-1*^*del-HDR*^ was higher compared to that of the control (normalized crossover points = crossover points / (neuron size × 10^−7^ μm^2^), 25.03 ± 8.24 in *RluA-1*^*−/−*^; *ppk-tdTom*, n = 6 *vs.* 13.48 ± 4.08 in *ppk-tdTom* control, n = 6, *P* < 0.05). Significant differences are marked as * (*P* < 0.05) and ** (*P* < 0.01). Data were analyzed with Student’s *t*-test. Error bars in all the figures represent S.D. (D). Representative traced dendritic structure of a class IV ddaC neuron of an L3 larva expressing *ppk-GAL4* > *UAS-td-Tom* in homozygous *RluA-1*^*del-HDR*^ (*RluA-1*^*−/−*^, left) in comparison with that of the *ppk-tdTom* control (+/+, right). The position of the cell body is marked with blue circle and axon with red circles.

A nociceptor-specific RNAi screen with thermal nociception assay discovered dozens of genes whose reduction caused either insensitive or hypersensitive thermal nociception (Honjo *et al.* 2016). Interestingly, some of those genes targeted with RNAi showed a reduced or increased branching of Class IV neuron dendrites. Reduced dendrite branching was often seen with nociceptive insensitivity while increased branching was found in some hypersensitive genotypes. Thus, regulation of Class IV neuron dendrite morphology is a commonly affected developmental pathway that is related to nociception phenotypes. Given this, we investigated the dendrite morphology of the cIVda neuron dendrites in the *RluA-1*^*del-HDR*^ mutant. In mutant ddaC neurons visualized with *ppk-CD4-tdTom*ato, we observed a modest but significant increase in the number of dendrite branches (normalized by neuron size) and shorter average branch length in comparison to control animals (Figures 8A and 8B). We also found that dendritic branches in the *RluA-1*^*del-HDR*^ ddaC neurons had higher frequency of isoneuronal cross-over events compared to the control (Figures 8C and 8D). This latter phenotype is suggestive of an isoneuronal tiling defect. Increased isoneuronal crossovers are also seen in mutants that affect dendrite attachment to the basal lamina (Han *et al.* 2012; Kim *et al.* 2012; Meltzer *et al.* 2016; Tenenbaum *et al.* 2017). Whether or not these dendrite abnormalities play a causal role in the hypersensitive nociception phenotypes of the *RluA-1* mutant will be an interesting subject for future investigation.

## Discussion

Given the well-established nociceptive role of md-neurons, we have investigated the historically first known molecular marker for md-neurons in nociception pathways. This gene encodes the RluA-1 protein in the RluA family of pseudouridine synthases. Our studies clearly demonstrate that loss of function for either *RluA-1* or *RluA-2* produce hyperalgesia in third instar *Drosophila* larvae. Tissue-specific RNAi, genetic null mutant, and cDNA rescue experiments all indicate that loss of the *RluA-1* gene from whole animals, or specifically from nociceptors, results in hyperalgesia. A newly generated *RluA-1*^*GAL4*^ driver showed specific expression in larval multidendritic and ES neurons. As well, a GFP exon trap for RluA-1 protein localized to the nuclei of these neurons. A small number of unidentified neurons in the larval brain were also revealed by *RluA-1*^*GAL4*^ and we observed expression of *RluA-1*^*GAL4*^ driven mCD8GFP and GFP tagged RluA-1 in cells of the adult brain, which included the optic lobe.

Although loss of *RluA-2* also caused hyperalgesia, its expression pattern was ubiquitous and included multidendritic neurons. Like the RluA-1 GFP exon trap, the RluA-2 GFP exon trap labeled nuclei. The nuclear localization for both RluA-1 and RluA-2 may indicate that these proteins act on RNA targets prior to export of the nucleus, or that they predominantly act upon nuclear localized RNAs. Structure based homology modeling and sequence alignments indicate that both RluA-1 and RluA-2 have 88% sequence similarity throughout their pseudouridine synthase domains. This evidence suggests that both of proteins are very likely to possess pseudouridine synthase activity but we have yet to formally prove this. In order to do so, we are working to identify potential target RNAs that could be used as substrates in experiments with purified RluA-1 and/or RluA-2 proteins.

We also observed that *RluA-1* mutants showed an increase in the number of dendrite branches relative to control genotypes as well as an increase in isoneuronal crossovers. Transcription factors, cytoskeletal regulators, motor proteins, secretory pathways and cell adhesion molecules all function in concert to develop and maintain optimum dendrite morphology (Corty *et al.* 2009). RluA-1 may modify RNAs for those genes that regulate dendritic morphology, or changes in the dendrite morphology could be an indirect consequence of neuronal sensitivity that is regulated by RluA-1. It is noteworthy that prior studies have noted a potential link between the degree of dendrite branching and the sensitivity of nociception behaviors (Honjo *et al.* 2016). In other cases, axonal factors such as the ion channel SK have been found to be important in regulating the cIVda neuron excitability (Onodera *et al.* 2017; Walcott *et al.* 2018). Whether the dendrite branching phenotype that we observe in *RluA-1* mutants is a cause or a consequence of hypersensitivity will be an interesting question for future studies.

A large body of literature indicates that RNA trafficking and local translation is important in dendrites and axons of neurons (Glock *et al.* 2017; Rangaraju *et al.* 2017). Relative to uridine the pseudourine base is believed to have enhanced rotational freedom which may alter conformation of RNA secondary structures. As well, an additional hydrogen bond donor present in pseudouridine may favor alternative base-pairing interactions in RNA. These properties may consequently alter RNA localization, stability and/or efficiency of translation (Arnez and Steitz 1994; Newby and Greenbaum 2002; Karikó *et al.* 2008). Pseudouridines can also influence decoding during translation as pseudouridylation of nonsense codons has been shown to suppress translation termination both *in vitro* and *in vivo* (Karijolich and Yu 2011). Thus, another possible function for pseudouridylation in nociceptive neurons could be to favor read-through of pseudouridylated stop codons to generate novel sequences at protein carboxy termini.

In summary, the data presented in this study showed the RNA pseudouridine synthases RluA-1 and RluA-2 are involved in nociception in *D. melanogaster*. The precise underlying mechanism can only be elucidated by identifying the RNA targets of these enzymes. Several groups have developed methods using next generation sequencing methods to identify the pseudouridine sites in transcriptomes (Carlile *et al.* 2014; Schwartz *et al.* 2014; Lovejoy *et al.* 2014; Li *et al.* 2015; Khoddami *et al.* 2019). We anticipate that future investigations applying these methods to wild type and *RluA* mutants in *Drosophila* will help us to identify the RluA targets.
